# The Teach-ABI Professional Development Module for Educators About Pediatric Acquired Brain Injury: Mixed Method Usability Study

**DOI:** 10.2196/43129

**Published:** 2023-05-15

**Authors:** Lauren Saly, Christine Provvidenza, Hiba Al-Hakeem, Andrea Hickling, Sara Stevens, Lisa Kakonge, Anne W Hunt, Sheila Bennett, Rhonda Martinussen, Shannon E Scratch

**Affiliations:** 1 Bloorview Research Institute Holland Bloorview Kids Rehabilitation Hospital Toronto, ON Canada; 2 Holland Bloorview Kids Rehabilitation Hospital Toronto, ON Canada; 3 Department of Occupational Science and Occupational Therapy Faculty of Medicine University of Toronto Toronto, ON Canada; 4 School of Rehabilitation Science McMaster University Hamilton, ON Canada; 5 Department of Speech-Language Pathology University of Toronto Toronto, ON Canada; 6 Rehabilitation Sciences Institute University of Toronto Toronto, ON Canada; 7 Department of Educational Studies Brock University St. Catherines, ON Canada; 8 Department of Applied Psychology and Human Development Ontario Institute for Studies in Education University of Toronto Toronto, ON Canada; 9 Department of Paediatrics University of Toronto Toronto, ON Canada

**Keywords:** acquired brain injury, educators, professional development, usability testing, satisfaction testing, knowledge translation, usability, death, disability, children, development, Ontario, research, online, school

## Abstract

**Background:**

Acquired brain injury (ABI) is a leading cause of death and disability in children and can lead to lasting cognitive, physical, and psychosocial outcomes that affect school performance. Students with an ABI experience challenges returning to school due in part to lack of educator support and ABI awareness. A lack of knowledge and training contribute to educators feeling unprepared to support students with ABI. *Teach-ABI*, an online professional development module, was created to enhance educators’ ABI knowledge and awareness to best support students. Using a case-based approach, *Teach-ABI* explains what an ABI is, identifies challenges for students with ABI in the classroom, discusses the importance of an individualized approach to supporting students with ABI, and describes how to support a student with an ABI in the classroom.

**Objective:**

This study aims to assess the usability of and satisfaction with *Teach-ABI* by elementary school educators. The following questions were explored: (1) Can elementary school teachers use and navigate *Teach-ABI*?, (2) Are the content and features of *Teach-ABI* satisfactory?, and (3) What modifications are needed to improve *Teach-ABI*?

**Methods:**

Elementary school educators currently employed or in training to be employed in Ontario elementary schools were recruited. Using Zoom, individual online meetings with a research team member were held, where educators actively reviewed *Teach-ABI*. Module usability was evaluated through qualitative analysis of think-aloud data and semistructured interviews, direct observation, user success rate during task completion, and the System Usability Scale (SUS) scores. The usability benchmark selected was 70% of participants performing more than half of module tasks independently.

**Results:**

A total of 8 female educators participated in the study. Educators were classroom (n=7) and preservice (n=1) teachers from public (n=7) and private (n=1) school boards. In terms of task performance, more than 85% of participants (ie, 7/8) independently completed 10 out of 11 tasks and 100% of participants independently completed 7 out of 11 tasks, demonstrating achievement of the module usability goal. The average overall SUS score was 86.25, suggesting a high satisfaction level with the perceived usability of *Teach-ABI*. Overall, participants found *Teach-ABI* content valuable, useful, and aligned with the realities of their profession. Participants appreciated the visual design, organization, and varying use of education strategies within *Teach-ABI*. Opportunities for enhancement included broadening content case examples of students with ABI and enhancing the accessibility of the content.

**Conclusions:**

Validated usability measures combined with qualitative methodology revealed educators’ high level of satisfaction with the design, content, and navigation of *Teach-ABI*. Educators engaged with the module as active participants in knowledge construction, as they reflected, questioned, and connected content to their experiences and knowledge. This study established strong usability and satisfaction with *Teach-ABI* and demonstrated the importance of usability testing in building online professional development modules.

## Introduction

### Background

Acquired brain injury (ABI) is defined as temporary or permanent damage to the brain that occurs after birth from a traumatic brain injury (TBI) or non-TBI [1]. ABI is a leading cause of death and disability in children [2]. After sustaining an ABI, outcomes vary based on several individual and injury-related factors, such as personality, preinjury strengths and needs, and location and severity of the injury [1-3]. Mild (ie, concussion), moderate, and severe brain injuries can lead to a variety of lasting cognitive, physical, and psychosocial outcomes [4-6] that affect students’ school performance [7,8].

Globally, educators report feeling underprepared and unaware of how to support students with ABI in the classroom [8-10]. School-aged children with ABI often experience challenges returning to school due in part to a lack of educator support and awareness of ABI [1]. Separate from an educators’ teaching approach, having ABI knowledge has been shown to influence academic and social domains for students [11]. Moreover, students with ABI report greater life satisfaction when their teachers are understanding of their needs and provide encouragement [11]. Therefore, a supportive school environment can facilitate successful school reintegration for children with ABI [12].

### The Canadian Context

In Canada, children with ABI are a “silent voice” in the education system [13]. Outside of exploring additional education or training specific to developmental disorders, educators do not receive adequate instruction related to ABI during preservice training or as practicing professionals [14]. For example, in Ontario, Canada’s most populated province, the Education Act (1980) [15] separates students with special education needs into 5 broad categories: intellectual, behavioral, communication, physical, and multiple. ABI is not a separate category, and unfortunately, the evolving nature of ABI may make it difficult to fit students’ areas of need into a distinct category [1]. For example, the diverse range of ABI symptoms includes a combination of cognitive, physical, psychosocial, and communication concerns. Hence, ABI is a unique exceptionality due to its wide, significant, and individualized impact across many domains of functioning [14]. Identification within a category equips educators with additional knowledge and awareness of strategies to support students within the Ontario education system. In 2018, the passing of Bill 193, also known as Rowan’s Law [16], mandated requirements to enhance concussion safety in Ontario. The act was created to raise awareness about concussion and improve concussion safety within amateur competitive sport by mandating sport organizations to (1) have athletes review concussion awareness and education resources approved by the Minister of Tourism, Culture and Sport; (2) develop a concussion code of conduct and have athletes review the code; and (3) establish a removal-from-sport and a return-to-sport protocol [16]. Many Ontario school boards responded to Rowan’s Law by implementing yearly concussion training for educators; however, evidence suggests that this training is brief and focuses on signs and symptoms, rather than addressing potential long-term impacts and how to support deficits [17]. Furthermore, concussion is only 1 condition under the diverse umbrella of ABI. Therefore, a gap in training related to mild, moderate, and severe ABI remains. Recently, Stevens and colleagues [10] confirmed that Ontario educators lack the knowledge and confidence to support students with ABI in the classroom. Ontario educators also reported the need for a course to improve their knowledge and awareness of pediatric ABI. Researchers at the Holland Bloorview Kids Rehabilitation Hospital (HBKRH) in Toronto, Ontario, responded to this need by creating an online professional development module called *Teach-ABI*.

### Development of Teach-ABI

#### Overview

The creation of *Teach-ABI* used an integrated knowledge translation (iKT) approach [18] and 2 process models throughout the design and testing phases: (1) Kern’s (2009) Six-Step Approach for Curriculum Development for Medical Education was used to develop *Teach-ABI* content and format [19]; and (2) the Knowledge-to-Action cycle [20] was used to consider the broader environment and context of this module. Importantly, Ontario educators were engaged as end users to co-design *Teach-ABI* to maximize usability and relevance in the education setting. Applying these process models resulted in 6 phases of module development: (1) problem identification, needs assessment, and an environmental scan; (2) curriculum development (eg, content and delivery); (3) usability testing; (4) pilot testing; (5) efficacy testing and preimplementation planning; and (6) sustainability planning and generalizability. This paper summarizes phases 1 and 2 of *Teach-ABI* development, and discusses the methodology and findings from the usability testing of *Teach-ABI* (phase 3). Phases 4-6 are planned as future work.

#### Phase 1: Problem Identification, Needs Assessment, and an Environmental Scan

The problem was identified and examined through a needs assessment workshop conducted with Ontario educators [10]. Educators confirmed the knowledge gap related to pediatric ABI and identified the need for a standardized, accessible, engaging, and short e-learning program that would help raise awareness and knowledge about pediatric ABI and the unique needs of these students in the classroom. An online format that incorporated a blended-learning approach, using instructional methods including videos and a case study, was suggested by educators [10]. A detailed environmental scan of publicly available resources was then conducted, with no existing resources meeting the identified need [21]. With this in mind, an interdisciplinary stakeholder group was formed to advise on the development of *Teach-ABI*. This stakeholder group included clinicians (eg, neuropsychologists, occupational therapist, speech language pathologist), researchers, a knowledge translation specialist, academic faculty of teacher’s colleges, teachers, and families and youth with lived experience of ABI.

#### Phase 2: Curriculum Development

The design of *Teach-ABI* involved defining specific and measurable learning objectives and developing educational strategies. Bloom’s Taxonomy [22] was used to inform the learning objectives of *Teach-ABI,* which focused specifically on fostering the remembering and understanding of information by end users. The established learning objectives of *Teach-ABI* were to (1) define ABI; (2) identify challenges for students with ABI in the classroom; (3) discuss the importance of taking an individualized approach to supporting students with ABI; and (4) describe how to support a student with an ABI in the classroom. These learning objectives formed the basis of the module content, which was developed by a practicing classroom teacher with specialized knowledge in pediatric ABI (LS). A knowledge-translation specialist (CP), an e-learning specialist, a graphic designer, and a videographer were engaged to develop the format of the module. *Teach-ABI* was created across multiple stages of iterative design and development in consultation with the interdisciplinary stakeholder group (2018-2019).

**Figure 1:**
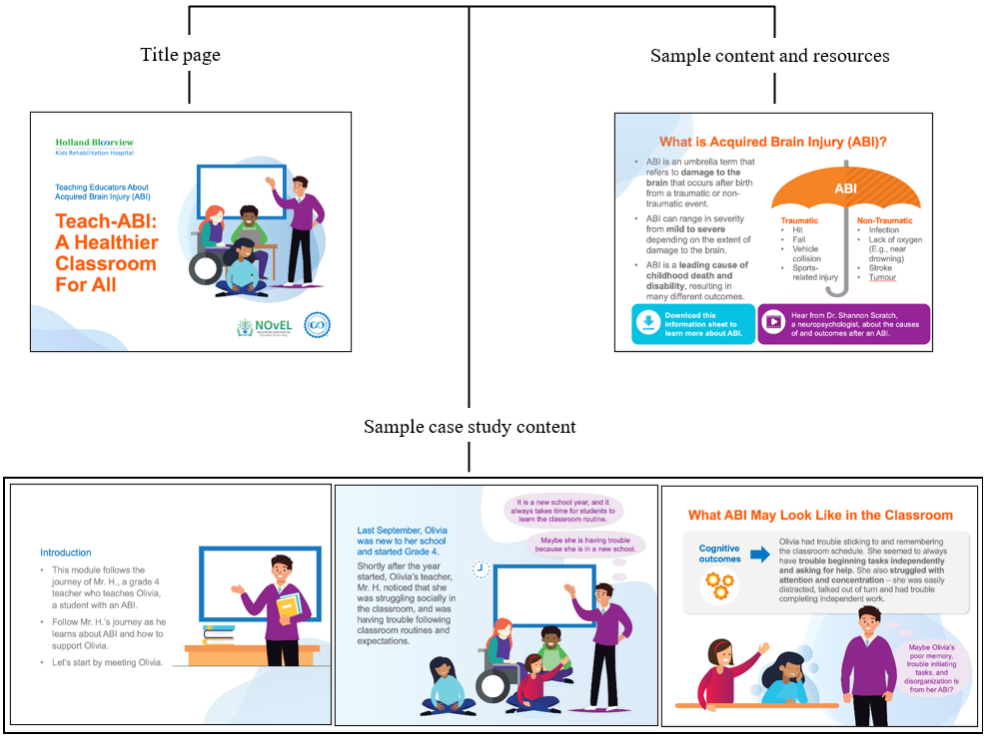
Screenshots of selected *Teach-ABI* components. ABI: acquired brain injury.

*Teach-ABI* is a self-directed, online module that provides information to educators about ABI causes and outcomes, and strategies for supporting students with ABI in the classroom. Given the broad developmental needs of students, the first iteration of *Teach-ABI* is designed for elementary school educators in Ontario, Canada. *Teach-ABI* introduces the concept of ABI and provides examples of potential challenges after an injury and appropriate strategies to support students in the classroom. *Teach-ABI* uses a case study design with links to websites and resources, embedded videos, and downloadable information sheets. The case study follows the story of Olivia, a grade 4 student who sustained an ABI at age 5, and Mr. H, her teacher, who learns how to support Olivia over time. *Teach-ABI* is divided into 2 parts: (1) an overview of ABI and the presentation of a student with an ABI in the classroom; and (2) ways to support a student with an ABI by providing classroom strategies for cognitive, emotional, physical, behavioral, and communication outcomes. See Figure 1 for screenshots showcasing different components of *Teach-ABI*.

#### Phase 3: Usability Testing

The primary focus of this study was to engage *Teach-ABI* end users, elementary school educators, to determine the usability of and satisfaction with the *Teach-ABI* module. Usability was conceptualized as a user’s experience with *Teach-ABI,* guided by questions used in previous investigations of online learning products: “Does the e-learning [resource] function as designed and intended?”; “Can learners interact with and navigate around as they need to?” [23]. This study focused on the perceived usability (ie, ease of use and navigation of the interface) and satisfaction (ie, subjective experience of end users) with the module design and content, as these aspects can affect users’ comprehension and application of information [24,25].

Given this, there were 3 main research questions:

- Can participants use and navigate *Teach-ABI*?
- Are the *Teach-ABI* content and features satisfactory?
- What modifications are needed to improve *Teach-ABI*?

## Methods

### Design and Participants

A mixed method prospective study design was used. Elementary educators were recruited including preservice teachers, classroom teachers, special education teachers, principals, vice principals, registered early childhood educators, and educational assistants. The authors acknowledge that the word *educator* is an umbrella term that encompasses teachers and other education-related professionals, the same way that the *school environment* is a term that includes the classroom and other aspects of school such as the playground. For the remainder of this paper, we will be using these terms interchangeably, in a similar fashion as our participants, to best reflect participant data.

Individuals were eligible to participate in the study if they met the following criteria: (1) currently enrolled in a teacher’s college program that will provide certification as an elementary school teacher with the Ontario College of Teachers; or (2) currently registered with the Ontario College of Teachers as an elementary school teacher (primary/junior or junior/intermediate teaching qualifications); or (3) currently working in an Ontario public elementary school as an educational assistant or registered early childhood educator. None of the interested participants met the following exclusion criteria: (1) non-English speaking; or (2) had cognitive, physical, or visual impairments that would require accommodations to use *Teach-ABI*. Community sampling through research flyers, social media, and the HBKRH website was used.

### Ethics Approval

Consent was obtained from all participants prior to commencing the study in compliance with the research ethics procedures (REB approval number 2020-0294-1588-2).

### Data Collection and Outcomes

Each participant attended a virtual private meeting over *Zoom* (Zoom Video Communications, Inc.), an online videoconferencing platform that has been utilized and found to be effective for facilitating qualitative data collection [26]. All questionnaires were hosted on REDCap (Vanderbilt University), a web-based platform for creating and managing surveys and survey data [27]. The usability of the *Teach-ABI* module was evaluated through qualitative analysis of think-aloud data and semistructured interviews, direct observation, user success rate during task completion, and the System Usability Scale (SUS) [28]. The think-aloud method originated from cognitive interviewing and invites participants to verbally share what they are thinking, feeling, and doing as they complete a task [29]. Topic probes were adapted from past health information usability studies [30]. Participants were instructed to comment on the module’s content, images, features, ease of interface use, aspects they liked or disliked, and suggestions for improvement.

While participants reviewed the module, they shared their screen in the *Zoom* meeting with the research team member. This allowed the researcher to capture the usability of the module through direct observation and evaluating task completion, which have been used to investigate the usability of online resources [30,31].

Field notes were taken as participants navigated the module and referenced content, images, and features. Notes were also included when participant verbalizations were vague (eg, “I really like this part”) or when any areas of difficulty or confusion arose. These notes were combined with the think-aloud data to examine the research questions.

The researcher observed participants’ completion of 11 tasks related to *Teach-ABI* (see Table 1 for the task list) and rated their level of success based on 1 of 3 outcomes: completed with ease, completed with help, and did not complete.

**Table 1:** *Teach-ABI*^a^ task list.

Task number	Task type
1	Access the module
2	Input information to create certificate
3	Browse content
4	Play the introduction video titled “Why the *Teach-ABI* Module Was Developed”
5	Download and open the tip sheet, titled “What Is Acquired Brain Injury”
6	Complete knowledge check (true or false)
7	Play the video titled “Supporting Students With ABI in the Classroom”
8	Hover over term to read definition of externalizing behaviors
9	Explore links outside the module and return back to the module
10	Navigating the module—return to previous slides
11	Access 1 or 2 resources in the resource list

^a^ABI: acquired brain injury

The chosen tasks were characteristic of actions that must be completed to successfully engage with the module. Participants were instructed to navigate the module as they normally would, which involved minimal to no interference from the researcher. Thus, participants were not asked to complete the specific tasks, rather, the researcher observed their completion of the tasks without any direction.

After reviewing the module, participants completed the SUS and a semistructured exit interview. The SUS is a validated 10-item questionnaire that provides a quick assessment of a system or tool’s perceived usability [32-34]. While the questionnaire was modified to suit this study (see Multimedia Appendix 1), research has demonstrated that minor linguistic changes do not impact the validity of the scale [32]. A semistructured interview guide was used to learn more about participants’ experiences completing *Teach-ABI*. Topics were consistent with previous usability studies and guidelines [35-37] and included overall impression, liked and disliked aspects of the module, navigation experience and feature usability, and suggestions for improvement. The exit interview was audio-recorded.

### Data Analysis

The average score on each of the 11 tasks was examined, in addition to participants’ individual scores on each task. A task was flagged as a usability problem if less than 70% of participants were able to complete it independently [30,38]. For this study, the usability goal was that more than half of the 11 tasks (ie, 6/11 tasks) would be completed independently by more than 70% (ie, n≥6/8) of participants. The ability to complete tasks needed to navigate *Teach-ABI* is a suitable way to determine module usability [39].

The SUS was scored using the steps outlined by Brooke [28]. Raw scores were converted to a total score out of 100. Scores were interpreted in relation to norm-referenced data, with an average score of 68 representing above average usability [33], and using a curved grading scale developed by Sauro and Lewis [40,41], which pairs scores out of 100 with a letter grade ranging from F (low) to A+ (high).

Two members of the research team (LS and HA-H) analyzed the qualitative data using content analysis [42,43]. Usability sessions and exit interviews were transcribed verbatim and reviewed multiple times for accuracy. Initial codes were generated from LS’s familiarization with the data and applied to the first transcript during line-by-line open coding. The initial list of codes was flexible and changed as the first transcript was coded. HA-H then coded the first transcript using the flexible list of codes. LS and HA-H discussed the codes and collaborated to clarify the existing codes and to create additional codes. These initial codes were used to code each transcript independently, and LS and HA-H met regularly to check for agreement related to the assigned codes and to create a final codebook. An explanation of each code was provided to ensure that they were applied consistently. Each code was also linked to one of the research questions to ensure the study focus remained central [44]. The codebook was flexible, as new codes were added throughout the coding process. Before a code was added, both researchers agreed on its inclusion and subsequent definition. The transcripts and codes were then organized in NVivo (QSR International), which was used to recode the transcripts based on the updated coding list. NVivo also provided structure and accessibility to the codes and meaning units within each code and allowed the data to be easily explored and reviewed to generate meaning and establish categories and subcategories [45]. The study reached data saturation as new or valuable information was not expected with additional interviews [46,47]. This is evident by the comprehensive information gathered when developing the categories and their relationships, as no new codes were identified following transcripts’ reviews.

## Results

### Participant Demographics

A total of 8 participants were enrolled in this study. The participants identified as female and were primarily early career practicing teachers employed by public school boards. Participants varied in their self-assessment of ABI knowledge. See Table 2 for participant information.

**Table 2:** Participant demographic characteristics.

Characteristic		Sample (N=8), n (%)
Gender, female		8 (100)
**Profession**		
	Classroom teacher	7 (88)
	Preservice teacher	1 (13)
**School employment setting**		
	Public	7 (88)
	Catholic	1 (13)
**Years of professional experience**		
	0-5	6 (75)
	6-10	0 (0)
	11-15	1 (13)
	16-20	0 (0)
	21-25	1 (13)
**Experience with students with ABI^a^**		
	Yes	4 (50)
	No	4 (50)
**Prior experience completing an e-learning module**		
	Yes	4 (50)
	No	4 (50)
**I feel that I have adequate knowledge about ABI**		
	Strongly disagreed/disagreed	4 (50)
	Neutral	2 (25)
	Strongly agreed/agreed	2 (25)

^a^ABI: acquired brain injury

### Task Performance

The average scores on each of the 11 tasks revealed that more than 85% (ie, 7/8) of participants independently completed 10 out of 11 tasks and 100% (8/8) of participants independently completed 7 out of 11 tasks. A usability problem occurred with downloading a tip sheet, with 5 participants able to independently download and open the tip sheet, 2 needing assistance, and 1 unable to complete this task. Overall, the study usability goal was met, as all participants completed more than 50% (>6/11) of tasks independently. See Table 3 for task performance scores.

**Table 3:** Task performance scores.

Task	Completed with ease (N=8), n (%)	Completed with help (N=8), n (%)	Did not complete (N=8), n (%)
Access the module	8 (100)	0 (0)	0 (0)
Input information to create certificate	7 (88)	0 (0)	1 (13)
Browse content	8 (100)	0 (0)	0 (0)
Play the introduction video titled “Why the *Teach-ABI*^a^ Module Was Developed”	7 (88)	1 (13)	0 (0)
Download and open the tip sheet, titled “What Is Acquired Brain Injury”	5 (63)	2 (25)	1 (13)
Complete knowledge check (true or false)	8 (100)	0 (0)	0 (0)
Play the video titled “Supporting Students With ABI in the Classroom”	8 (100)	0 (0)	0 (0)
Hover over term to read definition of externalizing behaviors	8 (100)	0 (0)	0 (0)
Explore links outside the module and return back to the module	8 (100)	0 (0)	0 (0)
Navigating the module—return to previous slides	8 (100)	0 (0)	0 (0)
Access 1 or 2 resources in the resource list	8 (100)	0 (0)	0 (0)

^a^ABI: acquired brain injury

### SUS

The average overall score on the SUS was 86.25 (range 65-100), surpassing the above average score for system usability (ie, 68) [33]. Using the curved grading scale [34,40,41], 86.25 translated into a score of A+.

### Qualitative Data

#### Overview

**Figure 2:**
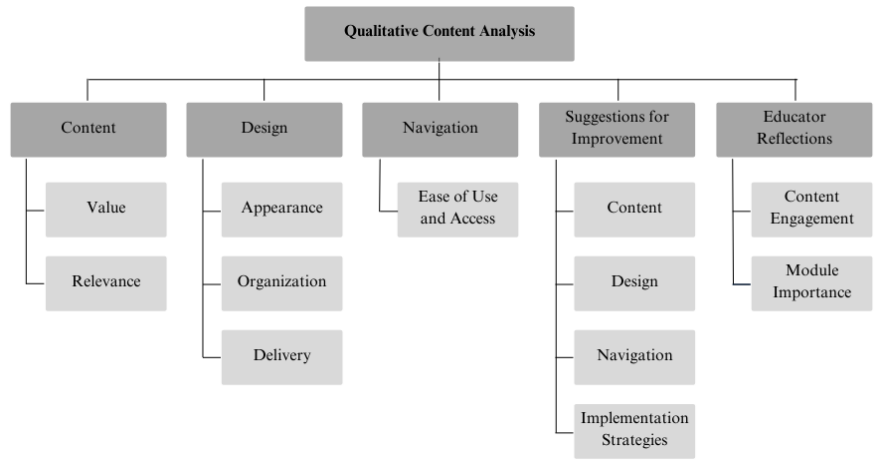
Categories and sub-categories from interview data.

Qualitative content analysis yielded 5 categories and 13 subcategories (Figure 2).

#### Content (Category 1): Value

Participants identified the module content as likable, understandable, and informative, and felt that the content was important to provide to educators. For example, one participant said, “Just talking about ABI in Ontario classrooms, I didn’t actually know that it’s not a recognized area of exceptionality and that it’s the leading cause of disability, so that is important for educators to know” [P6]. Similarly, participants shared that the content was informative, providing them with new knowledge: “This is new information to me. I just had no idea that there can be a delay in the challenges [after ABI]” [P2].

#### Content (Category 1): Relevance

As many as 7 participants felt the content was useful and aligned with the realities of their profession. For example, P2 stated “I found that it comes from a perspective where you understand the kids and you understand teachers and their perspective of the classroom.” Seven participants stated that they found the information to be useful, with 1 participant sharing their experience around downloading an information sheet, “Oh another sheet to download! I find these very useful” [P3].

#### Design (Category 2): Appearance

Participants found the module slides and tip sheets aesthetically pleasing (n=5) and enjoyed the colors (n=4) and pictures (n=4) used. For example, while navigating the module, participants shared: “I like the colour scheming so far” [P4] and “I like the use of pictures...It’s very visually appealing” [P6].

#### Design (Category 2): Organization

Participants appreciated that the module was divided into 2 parts: (1) ABI education and (2) supportive strategies (n=7). They also liked that the module had learning objectives and a content summary (n=5). They appreciated the use of bolding (n=5) and bullet points (n=6) to organize the slides, tip sheets, and videos. After reviewing the learning objectives, 1 participant shared, “I like the learning objectives. It really quantifies what am I going to get out of this module and makes it easier to make sure that I understand all of these steps by the time I’m done” [P5]. All participants appreciated the concision of the module components, expressing ideas such as, “I like that the videos are a reasonable amount of time” [P1]; “I like that [the information sheet is] short and easy to find the information” [P2]; “They’re to the point, easy to read, short, and won’t take up too much room on my computer” [P5].

#### Design (Category 2): Delivery

*Teach-ABI* presents information using various techniques, including interactive features (eg, reflection questions, knowledge check quizzes), videos, tip sheets, and the case study. Participants appreciated the varied techniques used and the engagement with the content that these techniques afforded. They expressed liking the case study approach to sharing information (n=6). When discussing the case study, participants stated: “The story of Olivia is great. It’s a nice way to follow something and to visualize it” [P4]; “I like the case study. It makes it more applicable and easier to understand” [P8]. Every participant shared their enjoyment of the knowledge check quizzes and a few highlighted that the quizzes made learning fun and accountable: “I did like the interactive pieces where you clicked to see the answer, or you dragged. Those things are fun!” [P4]; “I like the quizzes. They’re fun and they keep you accountable” [P7]. Participants enjoyed the lived experience videos (n=6) and described how the videos “humanize[d] the experience” [P5] and provided diverse perspectives of ABI (n=2).

#### Navigation (Category 3): Ease of Use and Access

The module was described as “user-friendly” (n=4), “easy to use,” (n=3), or “easy to follow” (n=3). Participants experienced little difficulty navigating the module and felt the features were simple to understand. They enjoyed the web-based nature of the module and its ease of use on personal devices: “I think a lot of modules I’ve used before open up in some weird flash player thing, so I liked that this was a web-based thing” [P8]. Participants found it easy to navigate between the slides and module sections (n=6), to access resource links that brought them outside the module, and to return to the module content: “I like that it’s hyperlinked so I can just easily access it” [P3].

#### Suggestions for Improvement (Category 4): Content

About one-third of the participants (n=3) suggested including additional examples of students with ABI to help broaden awareness of ABI and how it can affect children with different injuries; 2 participants shared a desire to learn more about the strategies listed to support students with ABI. Including resource links or videos that would provide further information about these strategies were suggested. For example, “I wish there was a resource that I could click there so I could learn more about that because that sounds interesting” [P8]. Participants expressed their enjoyment of the videos and the value they added to the module. Two participants frequently commented that it should be mandatory for participants to watch the videos with 1 sharing: “I think we should have to watch the videos” [P8].

#### Suggestions for Improvement (Category 4): Design

Two participants felt that the slides and text appeared small on their screen and suggested increasing font size, darkening the font, or creating a full-screen option that would expand the size of the slides. One participant noticed that the icon that invites users to download tip sheets was smaller than the other icons, making it seem less important. The same participant suggested making all of the icons the same size and adding an icon legend at the beginning of the module. Participants suggested reducing the amount of text on slides by using bullet points, charts, and images. One participant expressed, “A suggestion,...if [the slide] was bigger on the page, you could almost do a table with bullet points....often we deal with a lot of tables and bullet points...so it just becomes really easy to see” [P6]. Three participants mentioned adding a read-aloud feature, suggesting that it could improve users’ enjoyment of the module and meet educators’ different learning needs: “The one thing that I’ll say so far is that for some teachers, an audio feature, like listening to someone read it, would be really nice” [P6]. Participants commented on the length of time that it took to complete the module, sharing that most training completed at the beginning of the school year ranged from 15 to 30 minutes per module; yet the *Teach-ABI* module took longer to complete. They did not believe the length of time to complete the *Teach-ABI* module was unreasonable but emphasized the timing inconsistency in comparison to other training modules. They suggested reducing text on slides and including a read-aloud function to lessen the time taken to complete the module.

#### Suggestions for Improvement (Category 4): Navigation

All participants commented on the simple navigation of the module, referring to the module as “user-friendly,” “easy to use,” or “easy to follow.” Three participants had trouble navigating a pop-up arrow and as described in the “Design” subcategory above, noted small suggestions to improve functionality (eg, increasing font size, adding read-aloud function, reducing text amount).

#### Suggestions for Improvement (Category 4): Implementation Strategies

Participants suggested a variety of implementation strategies, methods for enhancing the uptake or implementation of a program [48], that could facilitate use of the *Teach-ABI* module by educators. One participant suggested including the module within a paid professional development course related to special education. These optional courses, called Additional Qualifications in Ontario, provide specialized information to teachers, such as how to support students with special education needs. Another participant felt that the detailed information in the module would be very informative for preservice teachers learning how the Ontario education system works to support students with disabilities, and should be a mandatory component of training. Finally, 1 participant suggested using special education resource teachers (SERTs) to disseminate information about ABI to their staff. The SERTs would receive additional training about ABI and could share the module with colleagues during an in-service professional development day and address questions educators may have about ABI.

#### Educator Reflections (Category 5): Content Engagement

Participants commented on the module content and its consistency with their beliefs and knowledge, and made connections to their experiences in the classroom. They also discussed ways to apply the information moving forward. After reading the introduction to the case study, 1 participant shared, “Now I’m curious about what happened. It’s like you opened a book for me and there’s a story and if I don’t go on it’s like closing the book in the middle, so I want to go on and find out what he did” [P2]. Teachers also put themselves in the case study educator’s shoes and shared what they would do in this situation. They appreciated that the knowledge checks and reflection questions made them stop and think about the information: “I like that it’s got a question that makes you think, because if you think about it according to Mr. H’s approach, a lot of it is what most teachers would do” [P5]. They also reflected on the content and how it aligned with their professional knowledge and experiences (n=5).

For some participants, interacting with the module content led to realizations about their previous experiences in the classroom. Two participants shared that the module made them think that they may have taught students with ABI before, but they were not aware of this at the time. One participant stated, “It gets me thinking about some kids that I totally missed the boat on, thinking ‘oh I wish I had known this before’” [P1], while another shared “I have many students that have been in these situations that play sports and now I’m sitting here thinking how many of them could have had this as well” [P6].

Some participants who had previously taught students with ABI reflected on how the module information aligned with their personal experiences. For example: “In my past relationships with ABI, it’s been that situation where it’s misdiagnosed...teachers get confused and it is easier to just stamp them with something that gets them an IEP versus, identifying what is ABI” [P5]. This level of reflection was not noted from teachers without previous experience working with students with ABI.

#### Educator Reflections (Category 5): Module Importance

Participants reflected on the importance of the *Teach-ABI* module in relation to their lack of awareness and related training, and the contribution of *Teach-ABI* to their knowledge of ABI. Five participants discussed the lack of awareness about ABI that exists among educators. For example, 1 participant shared, “I know when I talk to other teachers, I hear false things all the time about concussions and ABI – well concussions – we don’t know anything about ABI. There’s definitely a lot of confusion about ABI in the classroom” [P2]. They agreed that educators are not provided with adequate training related to ABI. Many participants felt the module had a positive impact on their knowledge of ABI and believed it should be accessible to other educators. One participant stated, “It achieved its goal of educating teachers on what ABI looks like in the classroom and what responses were effective, while also being considerate to the fact that everyone’s experience is so individualized that it’s going to be something you learn as you go, but this is kind of a basis for what you can expect” [P5]. Another shared, “I think it’s something that would really help a lot of educators, like there’s not a lot of information about it. I learned a lot about ABI. I didn’t know, I would say, any of that. I definitely think there’s a lack of knowledge in education and I think teachers need to have access to [*Teach-ABI*] in some form or another” [P6].

## Discussion

### Principal Findings

This study describes the development of *Teach-ABI* and outcomes of usability testing. The primary aim of this work was to assess ease of use of the *Teach-ABI* interface, determine end user satisfaction with the module design, and consider content modifications to optimize usability. The positive results expressed by the teacher participants regarding navigation of and satisfaction with the module maintain that *Teach-ABI* is a highly usable, professional development resource.

Use and navigation of *Teach-ABI* were assessed through data triangulation across multiple sources: participant observation and task performance, think-aloud data, the SUS, and exit interview data. Results demonstrated usability and ease of navigation of the *Teach-ABI* module. Participants completed 7 out of the 11 selected tasks at a rate of 100%. The average score on the SUS was 86.25, which is almost 20 points greater than scores awarded to systems with average usability [34]. Interview data also showed that participants described the module as easy to use and follow. Participant’s positive feedback on their experiences navigating the module contribute to an understanding of the module as user-friendly and highly learnable. Although a small number of participants encountered minor difficulties while navigating the module (eg, downloading the tip sheet, understanding pop-up arrow function), this can be remedied through minimal changes to the system.

In terms of *Teach-ABI* satisfaction, participants believed the module content was valuable, informative, and relevant to their profession. Module design, including the elements of appearance, organization, and delivery, was also rated positively. For example, participants appreciated the engaging features of the module (eg, case study, personal videos). Research highlights the importance of a module’s appearance and delivery, as it may influence users’ appeal and their ability to engage with the content [49]. Teacher participants also valued the self-reflection activities in *Teach-ABI*. Their reflections indicated that the module structure (eg, case study, knowledge checks) promoted a high level of user engagement and encouraged them to connect the topics to their practice.

The high level of satisfaction with the content and features of *Teach-ABI* speaks to the value of using an iKT approach combined with process models to inform product development [18-20]. Furthermore, the actions of reflecting on information, connecting it to previous experiences, and challenging existing knowledge link to higher-order thinking skills. This is significant, as eliciting higher-order thinking skills is associated with greater long-term recall and application of information [50]. In addition, educators were actively engaged with the module as they reflected on the content and made new connections to their personal teaching experience. Research has shown that reflection activities foster ongoing improvement in education practice and help to situate one’s context in their learning experience [51,52]. The inclusion of activities that end users can relate to through their teaching practice is a valuable design feature of online resources [51,53] and was the approach taken with the development of *Teach-ABI*.

### Considerations

Access to online professional development opportunities, such as *Teach-ABI*, does not ensure that educators have the knowledge needed to support students with ABI in the classroom. However, it is important to acknowledge that teacher participants identified the potential for *Teach-ABI* to improve their knowledge and understanding of ABI. This finding is promising and consistent with previous research on ABI training and its association with increased educator knowledge, fewer ABI misconceptions, and higher levels of confidence related to teaching students with ABI [54,55]. Furthermore, preliminary research has examined the effect of an online TBI training module on educators’ knowledge and confidence related to supporting students with TBI. The results indicated that the online module significantly improved educators’ knowledge and confidence related to supporting students with TBI and this improvement was maintained at the 30-day follow-up [9,56]. Guided by an iKT approach [18], next steps involve understanding facilitators and barriers to implementing *Teach-ABI*; supports needed to foster implementation; and the impact of the module on shifts in knowledge, confidence, and teaching practices. In addition, considering how educators with different backgrounds (eg, previous work with students with ABI, familiarity with e-learning modules, and level of ABI knowledge) experience and benefit from *Teach-ABI* is an important future direction.

### Strengths

The use of qualitative methods to examine usability was valuable, as it helped to explain the users’ response to content and features and situated their ratings of the module and suggestions for improving *Teach-ABI*. Besides, the sample size of the study is consistent with suggestions in the field of usability [57] and is suitable for reaching data saturation in qualitative interviewing [58]. Previous research studies examining training programs related to TBI have predominantly utilized quantitative methods, such as closed-ended surveys to examine usability [9,56], which provide a simple picture of usability. Qualitative methods helped achieve the program’s broader goal of creating a tool that is valuable and usable to Ontario educators and understanding participants’ experiences navigating *Teach-ABI*. In addition, the virtual data collection session resulted in an experience closely related to the real-world use of *Teach-ABI* by the study population. Instead of accessing *Teach-ABI* from a device provided by the researcher, participants accessed it using their own device and completed the module from a location of their choice, highlighting the ecological functionality of the module.

### Limitations

There are some limitations related to the study sample. For example, all 8 participants were practicing (n=7) or preservice (n=1) classroom teachers. Originally, the study aimed to recruit Ontario educators broadly, including practicing and preservice classroom teachers, principals, educational assistants, and early childhood educators, to extend the generalizability of the results. All 8 participants identified as female. Although there are a significantly greater number of Ontario elementary educators that identify as female, as many as 4 times more female elementary school teachers than male teachers [59], the sample was not representative of the teacher population. Furthermore, usability research suggests that males evaluate e-learning systems differently than females [60]; therefore, future research should aim to capture male educators’ perspectives on *Teach-ABI*. In addition, the sample consisted of mostly early career classroom teachers. It would be important to capture the perspective of teachers with more than 5 years of experience to understand any differences in their experience completing and navigating virtual modules in comparison to educators in the beginning stages of their careers. Lastly, the sample characteristics were limited due to self-selection bias. Participation in the study was voluntary and participants were recruited through sharing the research flyer and information on the social media and website of a research hospital. Future research should target a broader group of educators using a wider variety of recruitment methods.

### Conclusions

This study demonstrated strong usability and satisfaction with *Teach-ABI*, an innovative and novel online professional development module. Validated measures of usability combined with qualitative methodology revealed educators’ high level of satisfaction with the design, content, and navigation of *Teach-ABI*. Educators engaged with the module as active participants in knowledge construction, as they reflected, questioned, and connected content to their experiences and knowledge. This study highlights the importance of usability testing in the build of online professional development modules. Furthermore, the comprehensive approach to testing the usability of *Teach-ABI* may be applied in future studies evaluating online modules.

## Appendix

Multimedia Appendix 1 Adapted SUS statements. SUS: System Usability Scale.

## Abbreviations

ABI: acquired brain injury
HBKRH: Holland Bloorview Kids Rehabilitation Hospital
iKT: integrated knowledge translation
SERT: special education resource teacher
SUS: System Usability Scale
TBI: traumatic brain injury

## References

[1] Bennett S, Good D, Zinga D, Kumpf J (2004). Children with acquired brain injury: a silent voice in the Ontario school system. Exceptionality Education International.

[2] Thurman DJ (2016). The Epidemiology of Traumatic Brain Injury in Children and Youths: A Review of Research Since 1990. J Child Neurol.

[3] Ylvisaker M, Todis B, Glang A, Urbanczyk B, Franklin C, DePompei R, Feeney T, Maxwell NM, Pearson S, Tyler JS (2001). Educating students with TBI: themes and recommendations. J Head Trauma Rehabil.

[4] Bullock L, Gable R, Mohr J (2005). Traumatic Brain Injury: A Challenge for Educators. Preventing School Failure: Alternative Education for Children and Youth.

[5] Hawley C (2004). Behaviour and school performance after brain injury. Brain Inj.

[6] McKinlay A, Dalrymple-Alford J, Horwood L, Fergusson D (2002). Long term psychosocial outcomes after mild head injury in early childhood. J Neurol Neurosurg Psychiatry.

[7] Ewing-Cobbs L, Prasad MR, Kramer L, Cox CS, Baumgartner J, Fletcher S, Mendez D, Barnes M, Zhang X, Swank P (2006). Late intellectual and academic outcomes following traumatic brain injury sustained during early childhood. J Neurosurg.

[8] Linden MA, Braiden H, Miller S (2013). Educational professionals' understanding of childhood traumatic brain injury. Brain Inj.

[9] Glang A, McCart M, Slocumb J, Gau J, Davies S, Gomez D, Beck L (2019). Preliminary Efficacy of Online Traumatic Brain Injury Professional Development for Educators: An Exploratory Randomized Clinical Trial. J Head Trauma Rehabil.

[10] Stevens SA, Provvidenza C, Zheng S, Agnihotri S, Hunt A, Scratch SE (2021). Understanding the Needs of Ontario Educators in Supporting Students With Acquired Brain Injury in the Classroom. J Sch Health.

[11] Wlodarczyk K (2012). Educator evaluation of academic and social competence in students with acquired brain injury (ABI) relative to assessed performance and sense of belonging. Department of Graduate and Undergraduate Studies in Education, Faculty of Education, Brock University St. Catharines, Ontario.

[12] Parkin A, Maas F, Rodger S (1996). Factors contributing to successful return to school for students with acquired brain injury: parent perspectives. Australian Occupational Therapy Journal(3?4).

[13] Bennett S, Good D, Kumpf J (2003). Educating educators about acquired brain injury. Ontario Brain Injury Association.

[14] Zinga D, Good D, Kumpf J (2005). Policy and practice: acquired brain injury in Canadian educational systems. Canadian Journal of Educational Administration and Policy.

[15] Categories of exceptionalities | Part A: Legislation, policy and funding. Ontario Ministry of Education.

[16] Government of Ontario Rowan's Law (Concussion Safety) Law Document. Government of Ontario.

[17] Mallory KD, Saly L, Hickling A, Colquhoun H, Kroshus E, Reed N (2022). Concussion Education in the School Setting: A Scoping Review. J Sch Health.

[18] Gagliardi A, Berta W, Kothari A, Boyko J, Urquhart R (2016). Integrated knowledge translation (IKT) in health care: a scoping review. Implement Sci.

[19] Kern DE, Thomas PA, Hughes MT (2009). Curriculum Development for Medical Education: A Six-Step Approach (2nd edition).

[20] Graham Ian D, Logan Jo, Harrison Margaret B, Straus Sharon E, Tetroe Jacqueline, Caswell Wenda, Robinson Nicole (2006). Lost in knowledge translation: time for a map?. J Contin Educ Health Prof.

[21] Saly L, Marshall S, Mallory K, Hunt A, Kakonge L, Provvidenza C, Hickling A, Stevens S, Bennett S, Scratch S (2023). Pediatric acquired brain injury resources for educators: a multi-year scan of Canadian-relevant internet resources. Brain Inj.

[22] Bloom BS (1984). Taxonomy of Educational Objectives: Cognitive Domain.

[23] Phillips R, McNaught C, Kennedy G (2012). Evaluating e-Learning: Guiding Research and Practice.

[24] Gustafson D, Wyatt J (2004). Evaluation of ehealth systems and services. BMJ.

[25] Stinson J, McGrath P, Hodnett E, Feldman B, Duffy C, Huber A, Tucker L, Hetherington R, Tse S, Spiegel L, Campillo S, Gill N, White M (2010). Usability testing of an online self-management program for adolescents with juvenile idiopathic arthritis. J Med Internet Res.

[26] Archibald M, Ambagtsheer R, Casey M, Lawless M (2019). Using Zoom Videoconferencing for Qualitative Data Collection: Perceptions and Experiences of Researchers and Participants. International Journal of Qualitative Methods.

[27] Harris PA, Taylor R, Thielke R, Payne J, Gonzalez N, Conde JG (2009). Research electronic data capture (REDCap)-a metadata-driven methodology and workflow process for providing translational research informatics support. J Biomed Inform.

[28] Brooke J, Jordan PW, Thomas B, Weerdmeester BA, McClelland IL (1996). SUS: a "quick and dirty" usability scale. Usability Evaluation in Industry.

[29] Lewis C (1982). Using the "thinking-aloud" method in cognitive interface design.

[30] Barbara AM, Dobbins M, Haynes RB, Iorio A, Lavis JN, Raina P, Levinson AJ (2016). The McMaster Optimal Aging Portal: Usability Evaluation of a Unique Evidence-Based Health Information Website. JMIR Hum Factors.

[31] Cotton D, Gresty K (2006). Reflecting on the think-aloud method for evaluating e-learning. Br J Educ Technol.

[32] Bangor A, Kortum P, Miller J (2008). An Empirical Evaluation of the System Usability Scale. International Journal of Human-Computer Interaction.

[33] Brooke J (2013). SUS: a retrospective. Journal of Usability Studies.

[34] Lewis J (2018). The System Usability Scale: Past, Present, and Future. International Journal of Human–Computer Interaction.

[35] Georgsson M, Staggers N (2016). An evaluation of patients' experienced usability of a diabetes mHealth system using a multi-method approach. J Biomed Inform.

[36] Hernandez H (2018). The art of asking questions in usability testing. Akendi.

[37] Yalanska M (2020). Usability testing questions: tips and examples. Adobe.

[38] Rubin J, Chisnell D (2008). Handbook of Usability Testing: How to Plan, Design and Conduct Effective Tests.

[39] World Leaders in Research-Based User Experience (2017). Success rate: The simplest usability metric. Nielsen Norman Group.

[40] Lewis JR, Sauro J (2017). Can I Leave This One Out? The Effect of Dropping an Item From the SUS. Journal of Usability Studies.

[41] Sauro J, Lewis JR (2016). Quantifying the User Experience: Practical Statistics for User Research (2nd Edition).

[42] Hsieh H, Shannon SE (2005). Three approaches to qualitative content analysis. Qual Health Res.

[43] Sandelowski M (2000). Whatever happened to qualitative description?. Res Nurs Health.

[44] Elo S, Kyngäs Helvi (2008). The qualitative content analysis process. J Adv Nurs.

[45] Coffey A, Atkinson P (1996). Making Sense of Qualitative Data: Complementary Research Strategies.

[46] Brod M, Tesler LE, Christensen TL (2009). Qualitative research and content validity: developing best practices based on science and experience. Qual Life Res.

[47] Fusch P, Ness L (2015). Are we there yet? Data saturation in qualitative research. The qualitative report(9).

[48] Proctor EK, Powell BJ, McMillen JC (2013). Implementation strategies: recommendations for specifying and reporting. Implement Sci.

[49] Ritterband LM, Thorndike FP, Cox DJ, Kovatchev BP, Gonder-Frederick LA (2009). A behavior change model for internet interventions. Ann Behav Med.

[50] Jensen Jl, McDaniel Ma, Woodard Sm, Kummer Ta (2014). Teaching to the Test…or Testing to Teach: Exams Requiring Higher Order Thinking Skills Encourage Greater Conceptual Understanding. Educ Psychol Rev.

[51] Powell C, Bodur Y (2019). Teachers’ perceptions of an online professional development experience: Implications for a design and implementation framework. Teaching and Teacher Education.

[52] Scott DE, Scott S, Lindberg JO, Olofsson AD (2009). Innovations in the use of technology and teacher professional development. Online Learning Communities and Teacher Professional Development: Methods for Improved Education Delivery.

[53] Vrasidas C, Zembylas M (2004). Online professional development: lessons from the field. Education + Training.

[54] Ernst WJ, Gallo AB, Sellers AL, Mulrine J, MacNamara L, Abrahamson A, Kneavel M (2016). Knowledge of Traumatic Brain Injury among Educators. Exceptionality.

[55] Ettel D, Glang AE, Todis B, Davies SC (2016). Traumatic brain injury: persistent misconceptions and knowledge gaps among educators. Exceptionality Education International.

[56] McCart M, Glang AE, Slocumb J, Gau J, Beck L, Gomez D (2020). A quasi-experimental study examining the effects of online traumatic brain injury professional development on educator knowledge, application, and efficacy in a practitioner setting. Disabil Rehabil.

[57] Hwang W, Salvendy G (2010). Number of people required for usability evaluation. Commun. ACM.

[58] Ando H, Cousins R, Young C (2014). Achieving saturation in thematic analysis: development and refinement of a codebook. Comprehensive Psychology.

[59] Hoffman J (2017). A changing profession. Professionally Speaking.

[60] Ong C, Lai J (2006). Gender differences in perceptions and relationships among dominants of e-learning acceptance. Computers in Human Behavior.

