# Supplementary material for: The Teach-ABI Professional Development Module for Educators About Pediatric Acquired Brain Injury: Mixed Method Usability Study
Source: JMIR Hum Factors. 2023 May 15;10:e43129. doi: 10.2196/43129 (PMC10227698; doi:10.2196/43129)
Supplement: Multimedia Appendix 1 [file humanfactors_v10i1e43129_app1.docx]

Appendix A:

Adapted SUS Statements

| **Original SUS Statements [28]** | ****  **Adapted SUS Statements**  **** |
| --- | --- |
|  |  |
| 1) I think that I would like to use this system frequently | 1) I think that I would like to use this module frequently |
| 2) I found the system unnecessarily complex | 2) I found this module unnecessarily complex |
| 3) I thought the system was easy to use | 3) I thought this module was easy to use |
| 4) I think that I would need the support of a technical person to be able to use this system | 4) I think that I would need assistance to be able to use this module |
| 5) I found that the various functions in this system were well integrated | 5) I found that the various functions in this module were well integrated |
| 6) I thought that there was too much inconsistency in this system | 6) I thought that there was too much inconsistency in this module |
| 7) I would imagine that most people would learn to use this system very quickly | 7) I would imagine that most people would learn to use this module quickly |
| 8) I found the system very cumbersome to use | 8) I found this module very cumbersome/awkward to use |
| 9) I felt very confident using the system | 9) I felt very confident using this module |
| 10) I needed to learn a lot of things before I could get going with this system | 10) I needed to learn a lot of things before I could get going with this module |
